# Imaging findings of inflammatory myofibroblastic tumor of sigmoid colon: literature review and case report

**DOI:** 10.3389/fmed.2024.1461205

**Published:** 2024-08-13

**Authors:** Xianwen Hu, Wei Zhao, Ronghua Yu, Pan Wang

**Affiliations:** ^1^Department of Nuclear Medicine, Affiliated Hospital of Zunyi Medical University, Zunyi, China; ^2^Department of Pathology, Affiliated Hospital of Zunyi Medical University, Zunyi, China

**Keywords:** inflammatory myofibroblastic tumor, sigmoid colon, ^18^F-FDG, PET/CT, CT

## Abstract

Inflammatory myofibroblastic tumor (IMT) is an intermediate tumor composed of differentiated myofibroblastic spindle cells with inflammatory cell infiltration. It can occur in all parts of the body, with the lungs being the most common, while the tissues outside the lungs, including the sigmoid colon, are rare. Herein, we present a case of a 10-year-old girl with sigmoid IMT who presented to our hospital with abdominal pain. An abdominal computed tomography (CT) revealed a well-defined, slightly low-density mass in her lower abdomen that was not clearly demarcated from the sigmoid colon. The mass showed significant uneven enhancement on contrast-enhanced CT and increased fluorine-18 fluorodeoxyglucose (^18^F-FDG) uptake on positron emission tomography (PET). Moreover, a systematic review of the published literature on sigmoid IMT was conducted and its clinical and radiographic features were summarized to increase the understanding of this rare disease.

## Introduction

Inflammatory myofibroblastic tumor (IMT) is a rare intermediate mesenchymal tumor, which has previously been described as inflammatory pseudotumor, plasma cell granuloma, and inflammatory myofibrous histiocytic proliferation, consisting of differentiated myofibroblastic spindle cells, often accompanied by extensive lymphocyte and/or plasma cell infiltration (1). The etiology of IMTs is unknown and may be related to certain special bacterial or EB virus infections, chromosomal mutations (2). It can be seen at any age, but is mainly found in children and young adults, with females being more common (3). The tumor can occur in various parts of the body, of which the lung is the most common, accounting for 95%, while the organ tissues outside the lung, including mesentery, omentum, liver, retroperitoneum and limbs, are rare (4). The disease symptoms of patients are related to the disease site, including fever, pain, anemia and mass, without specificity (5). It is precisely because of the rarity of IMTs and the non-specificity of clinical manifestations that it is difficult to make a correct diagnosis before surgery. Here, we present the diagnosis and treatment of a rare patient with sigmoid IMT and review the literature with a view to increasing awareness of this rare disease.

## Case presentation

**Figure 1:**
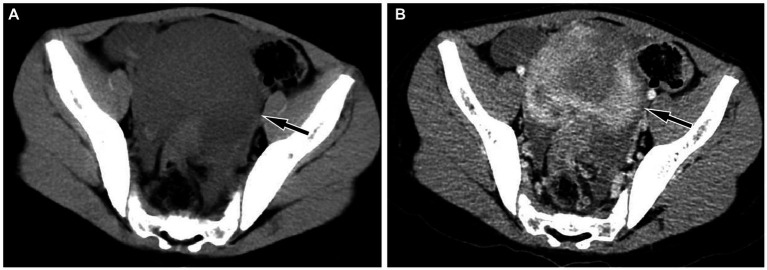
Abdominal CT revealed a regular low-density mass (**A**, arrow) in the pelvic cavity, about 9.3 cm × 9.0 cm × 6.7 cm in size; which presented significant uneven enhancement on contrast-enhanced CT (**B**, arrow).

**Figure 2:**
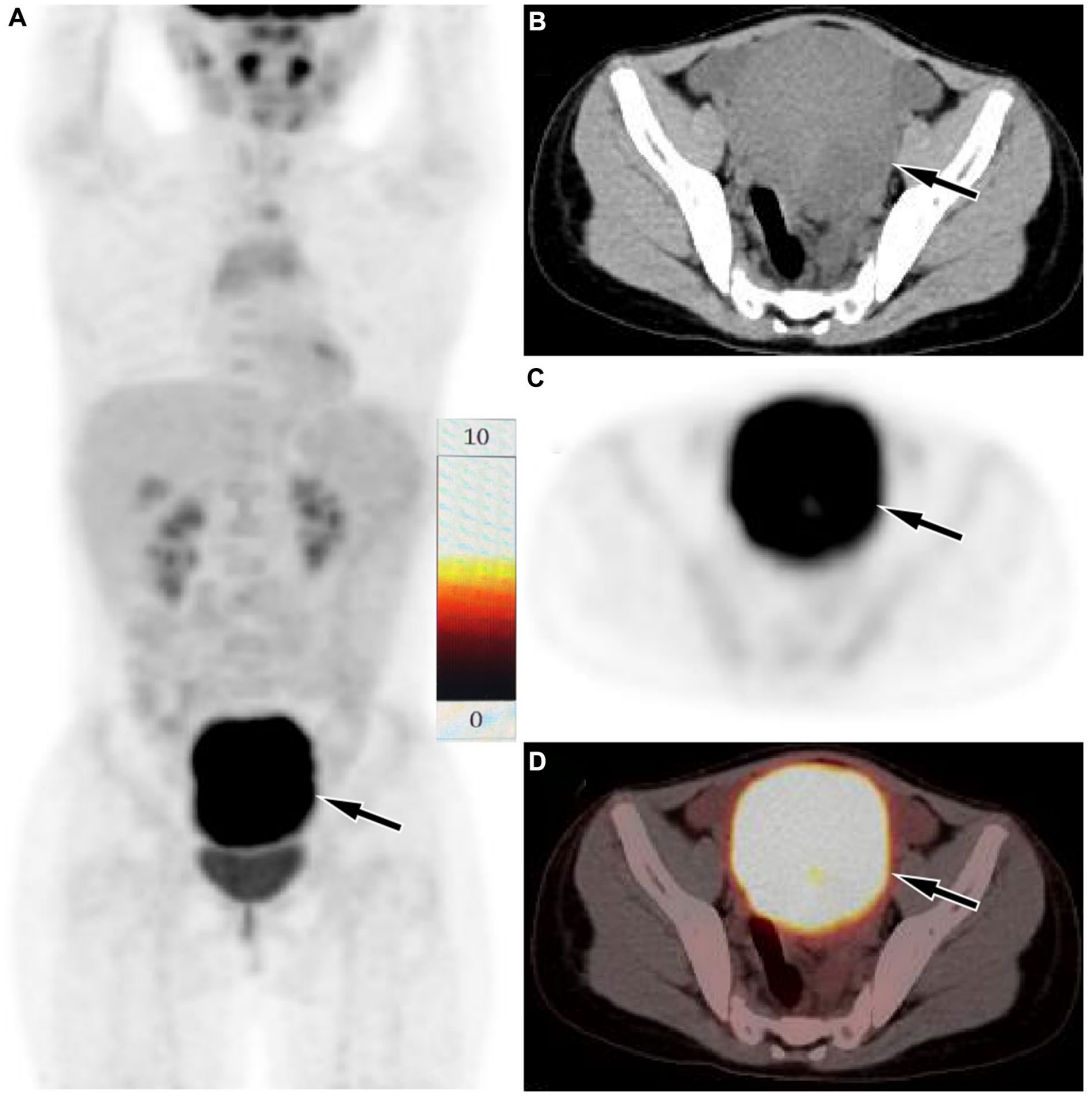
Fluorine-18 fluorodeoxyglucose (^18^F-FDG) positron emission tomography (PET)/CT imaging of the patient. The maximum intensity projection (MIP, **A**) showed a significantly increased ^18^F-FDG uptake in the lower abdominal area (arrow). Axial CT **(B)**, PET **(C)** and PET/CT fusion **(D)** showed that the lesion with significantly increased ^18^F-FDG uptake, with a SUVmax of 9.3 (arrows), which was located in the serosal surface of the sigmoid colon, and the adjacent intestinal tube is compressed, the proximal intestinal tube is dilated, and liquid exudation shadow is seen around the mass, and a small amount of fluid is seen in the pelvic cavity.

**Figure 3:**
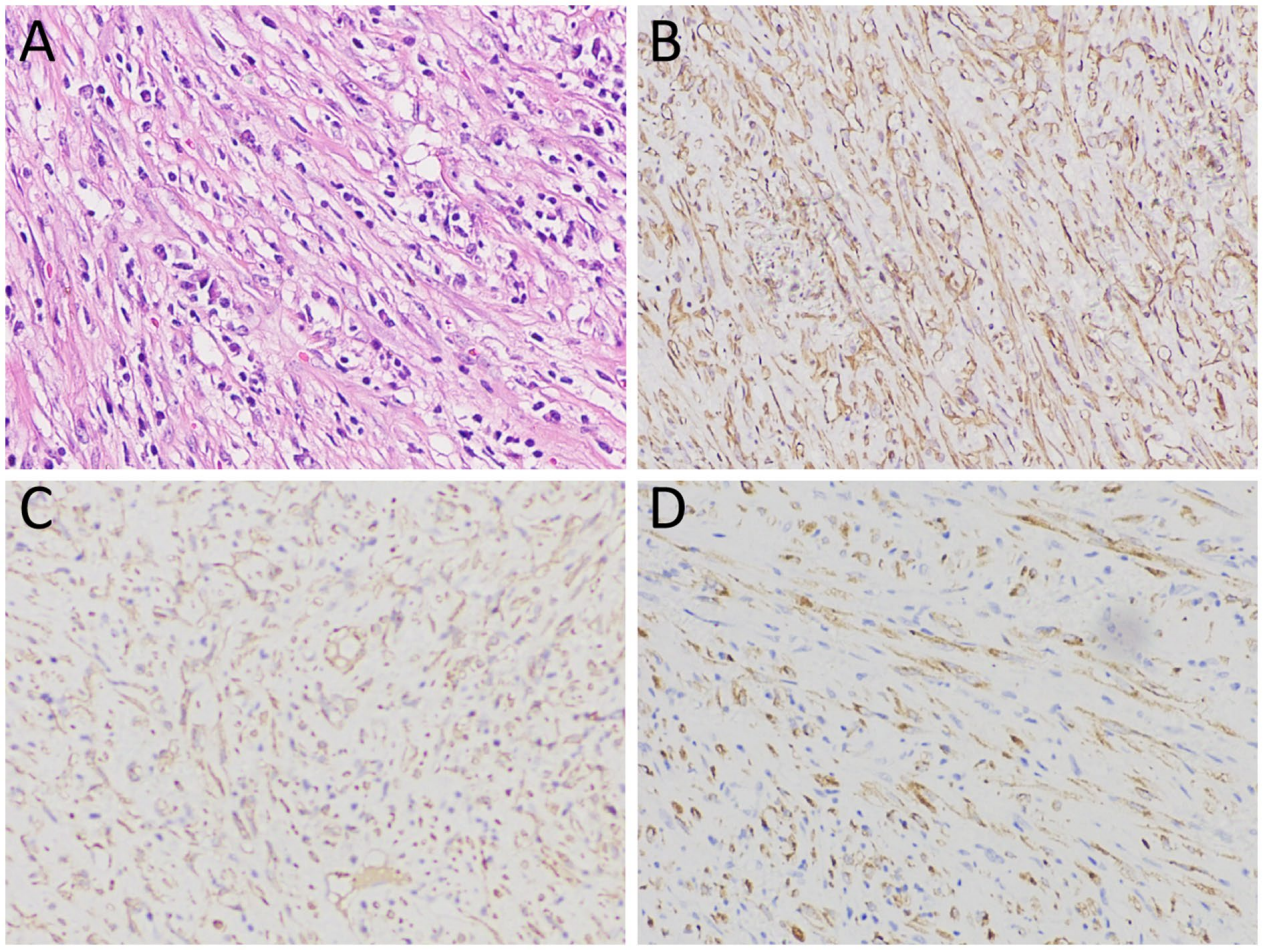
**(A)** Hematoxylin–eosin staining revealed diffuse spindle shaped tumor cells and scattered inflammatory cells within the lesion; Immunohistochemical results showed that the tumor cells positively expressed SMA **(B)**, ALK **(C)** and vimentin **(D)**. Notes: SMA, smooth muscle actin; ALK, anaplastic lymphoma kinase.

A 10-year-old girl with abdominal pain for 3 days underwent an abdominal ultrasound examination in an outside hospital on November 20, 2023 and found a large mass in her pelvic cavity. She was admitted to our hospital on 23 November 2023 for further diagnosis and treatment. She and her family denied any history of tumors or genetic diseases. Physical examination revealed a large, hard, low-motion mass palpable in her pelvic cavity, while no significant positive signs were found in the rest of her body. The laboratory test results, including serum tumor markers, were all negative. On November 24th, the patient underwent an abdominal CT examination (Figure 1) and the results showed a slightly low-density mass with unclear boundary with the sigmoid colon in her pelvic cavity, which presented significant uneven enhancement on contrast-enhanced CT, suggesting a possible malignant tumor. In order to further evaluate the nature of the tumor and determine the treatment plan, she underwent ^18^F-FDG PET/CT examination the following day. The results showed obviously increased ^18^F-FDG uptake in this lesion, and no hot spot lesions were observed in the rest of the body (Figure 2). Based on these imaging findings, the patient was initially suspected to have a malignant lesion. After a series of evaluations, the patient underwent an exploratory laparotomy, radical tumor resection and ileostomy on November 29 under anesthesia. Hematoxylin–eosin staining revealed diffuse spindle shaped tumor cells and scattered inflammatory cells in resected tumor tissue (Figure 3). Immunohistochemical results showed that the tumor cells positively expressed smooth muscle actin (SMA), anaplastic lymphoma kinase (ALK), CD117, vimentin, but negatively expressed cytokeratin (CK), Desmin and Dog-1. Based on these histopathological features, the patient was diagnosed with an inflammatory myofibroblastic tumor. The patient improved after receiving 3 days of anti-inflammatory treatment with ceftriaxone after surgery and was discharged on December 1, 2023. On June 2, 2024, she underwent abdominal ultrasound examination and showed no signs of tumor recurrence. The patient has been following up for 6 months now and has not reported any discomfort.

## Literature review

The PubMed and Web of Science databases were searched for case reports and series of sigmoid colon IMT as of June 10, 2024, with language limitations in English. The search strategy was as follows: (“inflammatory myofibroblastic tumor” OR “inflammatory pseudotumor” OR “plasma cell granuloma” OR “inflammatory myofibrous histiocytic proliferation”) AND “sigmoid colon.” For each enrolled case, the first author, publication year, as well as the patient’s gender, age, clinical symptoms, imaging findings including CT and PET, and follow-up results were recorded (Table 1).

**Table 1:** Clinical and imaging features of the cases of sigmoid colon sigmoid colon IMT from the literature review and current case.

Case, no.	Author/year/country	Gender /Age (years)	Symptom	CT imaging			PET (SUVmax)	Treatment	Follow-up (month)
				Morphological changes	MD (cm)	CECT			
1(6)	Dhuria S/ 2018/India	F/2	vomiting, fever,	oval, isodense solid soft tissue mass with low-density necrosis	8.0	OUE	NA	Surgery	36/NR
2 (7)	Pandit N/ 2019/Nepal	M/35	blood mixed stool, fever, weight loss	lobulated isodense soft tissue mass	8.0	OEE	NA	Surgery	12/NR
3 (8)	Chinnakkulam KS/2020/India	F/40	abdominal pain, vomiting, constipation	suborbicular isodense soft tissue mass	6.0	OEE	NA	Surgery	18/NR
4 (9)	Kavirayani V/ 2023//India	F/9 months	abdominal distension	suborbicular isodense soft tissue mass with low-density necrosis	9.4	OUE	NA	Surgery	6/NR
5 (10)	Wu L/2023 /China	M/11 months	vomiting, fever,	lobulated cystic solid mass	12.0	OUE	NA	Surgery	24/NR
6 (12)	Uysal S/2005 /Turkey	M/11	abdominal pain, difficult defecation	suborbicular isodense soft tissue mass with low-density necrosis	11.0	OUE	NA	Surgery	6/NR
7 (11)	Nakamura Y/ 2010/Japan	F/82	abdominal pain	NA	NA	NA	NA	Conservative treatment	24/NR
8	Present case	F/10	abdominal pain	suborbicular low-density mass	9.3	OUE	15.4	Surgery	6/NR

IMT, inflammatory myofibroblastic tumor; MD, maximum diameter; PET, positron emission tomography; SUVmax, maximum standard uptake value; CT, computed tomography; CECT, contrast-enhanced computed tomography; OUE, obvious uneven enhancement; OEE, obvious even enhancement; NR, no recurrence; NA, not applicable.

After a systematic search and careful reading of the full text of the preliminary screening, it was finally determined that there were seven cases of sigmoid IMT published before our case (6–12). Including the current patient that we reported, a total of eight sigmoid IMT cases, consisting of three male (3/8) and five female (5/8) patients, with a median age of 10.5 years (range, 9 months-81 years old), were included in the analysis. Common clinical symptoms include abdominal pain, vomiting, fever, and abdominal discomfort. IMTs are generally large in size, with a mean maximum diameter of 9.1 cm. Most of the patients (7/8) underwent surgery, and only one patient received conservative treatment due to poor lung function. The prognosis of IMT was good, and no significant signs of tumor recurrence were found in all patients who underwent surgical resection of the mass during the follow-up period.

## Discussion

IMT in the sigmoid colon is rare. Our current study presents a case of a child diagnosed with sigmoid IMT who complained of abdominal pain. To further understand the characteristics of this disease, we conducted a systematic review of relevant literature, and the results showed that most IMT patients in the sigmoid colon were children and young adults, with more females than males. This is consistent with the epidemiology of IMT patients occurring in other parts of the body (13). Most patients seek medical help due to vomiting, fever, abdominal pain, and abdominal discomfort. The literature reported that the etiology of IMT may be related to chronic infections, autoimmune diseases, and trauma (2). However, our case and previous literature on IMT in the sigmoid colon have not reported any such medical history, so this viewpoint may need to be confirmed in the future.

Imaging examinations play a significant role in the diagnosis of IMT, and the imaging of IMT in the sigmoid colon has certain characteristics. On CT, it usually presents as a large isodense soft tissue mass with smooth edges, and there may be low-density cystic necrosis area within the mass. On contrast-enhanced CT, the mass showed obvious uniform or uneven enhancement (6–10). Unlike previous literature reports, the current case showed a uniform low-density mass on CT, but still showed significant enhancement on contrast-enhanced CT. At present, research on the ^18^F-FDG/glucose metabolism of IMT is relatively rare and is mostly seen in case reports. Most IMTs present significantly increased ^18^F-FDG uptake on PET and are often misdiagnosed as malignant tumors in the corresponding area (14–16). The mechanism of ^18^F-FDG uptake by IMT may be correlated with tumor cellularity, inflammatory cell infiltration and Ki-67 expression. The higher tumor cellularity, more composition of inflammatory cell and higher Ki67 expression, the greater SUVmax (17). The patient we reported presented with low density on CT, with exudative shadows around the mass and obvious uptake of ^18^F-FDG on PET, which may be related to the strong inflammatory cell infiltration of the mass. To our knowledge, our case study is the first to report PET findings of sigmoid colon IMT, which, like IMT occurring in other organ tissues, also showed significantly increased ^18^F-FDG uptake.

According to the imaging findings of IMT, IMT originating from the sigmoid colon needs to be differentiated from gastrointestinal stromal tumors (GISTs), lymphoma and sigmoid colon cancer. GISTs also presented as large, circular or lobulated soft tissue masses on CT, with cystic necrosis at the center of the mass and uneven delayed enhancement on contrast-enhanced CT (18). On PET, GISTs with different risk levels show varying levels of increased ^18^F-FDG uptake, and high-risk GISTs presenting a higher SUVmax than medium-to low-risk GISTs (19). Moreover, due to the fact that GISTs typically grow outside the intestinal lumen, GISTs located in the lower abdomen may exhibit migration of the mass over time on dual-time point PET/CT, which is a relatively specific sign (20). Lymphomas that occur in the intestine are mostly B-cell non-Hodgkin lymphomas. Like IMT, it presents significantly increased ^18^F-FDG uptake on PET (21). However, intestinal lymphoma usually infiltrates along the intestinal wall, presenting as a circular thickening of the intestinal wall, and rarely causing proximal intestinal duct dilation and obstruction (22). Adenocarcinoma is the most common tumor in the sigmoid colon, and it also shows significantly increased ^18^FDG uptake on PET. However, sigmoid colon cancer often grows infiltratively along the intestinal wall and has an irregular shape on CT (23), which is significantly different from IMT.

Pathological examination is currently the gold standard for diagnosing IMT. Microscopically, the characteristic fusiform myofibroblast proliferation was observed, accompanied by abundant infiltration of chronic inflammatory cells such as plasma cells, T lymphocytes, neutrophils, and eosinophils (24). Immunohistochemistry showed that tumor cells positively expressed vimentin, SMA, and Desmin usually, and CK, CD68, CD30 and ALK were partially expressed positively, while CD117 and CD34 were usually negative expressed (25). In the tumor tissue of the patient we reported, diffuse fusiform tumor cells and scattered inflammatory cells were found in the lesion under microscope. Immunohistochemical results showed that the tumor cells positively expressed vimentin, SMA, and ALK, which was consistent with the pathological diagnosis of IMT.

At present, radical surgical resection of tumor tissue is the preferred treatment for IMT. Only when the tumor cannot be surgically removed, other treatment options including chemotherapy, immunomodulatory therapy, corticosteroids, radiotherapy, nonsteroidal anti-inflammatory drugs and so on should be considered (26, 27). Since IMT is an intermediate tumor with the possibility of recurrence and metastasis, close and careful follow-up after complete surgical resection of the tumor tissue is crucial to improve the prognosis of patients (28). The patient we reported did not show any signs of tumor recurrence or metastasis during follow-up after surgical removal of the tumor.

In conclusion, sigmoid IMT is a relatively rare intermediate tumor and should be considered in the differential diagnosis of other sigmoid malignancies such as GISTs and cancers. It appears as a large, smooth edge, or low-density mass on CT, with obvious uniform or uneven enhancement on contrast-enhanced CT, which showed significantly increased ^18^F-FDG uptake on PET. These imaging findings contribute to the diagnosis of IMT.

## Data availability statement

The original contributions presented in the study are included in the article/supplementary material, further inquiries can be directed to the corresponding author.

## Ethics statement

Written informed consent was obtained from the individual(s), and minor(s)’ legal guardian/next of kin, for the publication of any potentially identifiable images or data included in this article.

## Author contributions

XH: Conceptualization, Data curation, Formal analysis, Funding acquisition, Writing – original draft. WZ: Investigation, Methodology, Project administration, Writing – original draft. RY: Conceptualization, Methodology, Validation, Writing – original draft. PW: Investigation, Project administration, Supervision, Visualization, Writing – review & editing.
